# FTY720 Induces Apoptosis of M2 Subtype Acute Myeloid Leukemia Cells by Targeting Sphingolipid Metabolism and Increasing Endogenous Ceramide Levels

**DOI:** 10.1371/journal.pone.0103033

**Published:** 2014-07-22

**Authors:** Limin Chen, Liu-Fei Luo, Junyan Lu, Lianchun Li, Yuan-Fang Liu, Jiang Wang, Hong Liu, Heng Song, Hualiang Jiang, Sai-Juan Chen, Cheng Luo, Keqin Kathy Li

**Affiliations:** 1 State Key Laboratory of Medical Genomics, Shanghai Institute of Hematology, Rui Jin Hospital Affiliated to Shanghai Jiao Tong University School of Medicine, Shanghai, China; 2 Drug Discovery and Design Center, State Key Laboratory of Drug Research, Shanghai Institute of Materia Medica, Chinese Academy of Sciences, Shanghai, China; 3 Department of Chemistry, East China University of Science and Technology, Shanghai, China; University of Louisville, United States of America

## Abstract

The M2 subtype Acute Myeloid Leukemia (AML-M2) with t(8;21) represents an unmet challenge because of poor clinical outcomes in a sizable portion of patients. In this study,we report that FTY720 (Fingolimod), a sphingosine analogue and an FDA approved drug for treating of multiple sclerosis, shows antitumorigenic activity against the Kasumi-1 cell line, xenograft mouse models and leukemic blasts isolated from AML-M2 patients with t(8;21) translocation. Primary investigation indicated that FTY720 caused cell apoptosis through caspases and protein phosphatase 2A (PP2A) activation. Transcriptomic profiling further revealed that FTY720 treatment could upregulate AML1 target genes and interfere with genes involved in ceramide synthesis. Treatment with FTY720 led to the elimination of AML1-ETO oncoprotein and caused cell cycle arrest. More importantly, FTY720 treatment resulted in rapid and significant increase of pro-apoptotic ceramide levels, determined by high-performance liquid chromatography-electrospray ionization tandem mass spectrometry based lipidomic approaches. Structural simulation model had also indicated that the direct binding of ceramide to inhibitor 2 of PP2A (I2PP2A) could reactivate PP2A and cause cell death. This study demonstrates, for the first time, that accumulation of ceramide plays a central role in FTY720 induced cell death of AML-M2 with t(8;21). Targeting sphingolipid metabolism by using FTY720 may provide novel insight for the drug development of treatment for AML-M2 leukemia.

## Introduction

Acute myeloid leukemia (AML)is a heterogeneous clonal disorder of the myeloid line of blood cells and is characterized by the accumulation of immature myeloid progenitors in the bone marrow and peripheral blood. Clinical outcomes are particularly poor for AML subtypes with contain chromosomal abnormalities, such as t(8;21) translocation, which accounts for 40% to 80% of M2 type AML (AML-M2) [1].AML1-ETO fusion proteinis generated by t(8∶21) translocation [2], which is thought to be a transcriptional repressor of AML1 target genes. The median survival time of these patients is no more than two years and the five-year survival rate is less than 40% [3]. Currently, cytosine arabinoside (Ara-C) based chemotherapy is one of the standard induction therapies for AML patients. However, since Ara-C is a nucleoside analog, high dosages cause a number of undesirable side effects. Thus, despite the conventional chemotherapeutic approaches, there is an urgent need to identify novel anti-tumor agents for treating AML-M2 with new mechanisms and low toxicity.

As an immunosuppressive agent, FTY720 (2-amino-2-[2-(4-*n*-octylphenyl)ethyl]-1, 3-propanediol hydrochloride) is synthetically derived from myriocin (ISP-1), a metabolite isolated from fungus *Isaria Sinclairii*, and was approved by the US Food and Drug Administration (FDA) for treatment of multiple sclerosis in 2010 [4]. FTY720 has previously shown activity in several hematologic malignancies [5]–[10]. In another study,FTY720demonstrated the potential toincrease the radio therapeutic sensitivity of prostate cancer cells and reduce tumor growth and metastasis without toxic side effects [11].Despite the extensive investigations intothe anti-cancer effects of FTY720, the mechanism of FTY720 induced cell death is disputed, asFTY720 shows an apparent heterogeneous effectsacross different hematologic malignancies. FTY720 could induce apoptosis or non-apoptotic cell death through either caspase dependent or independent pathways [5], [6], [12]–[14]. Meanwhile, activation of PP2A has been considered to be a common event in FTY720 induced cell death [5], [6], [12], [14]. Other distinct mechanisms for FTY720 induced cell death, such as down regulating nutrient transporter proteins [15],inducing reactive oxygen species (ROS) [16], and activating sterol regulatory element-binding protein (SREBP) [17] have also been proposed.

Herein, we report that FTY720 has potent *in vitro* and *in vivo* antitumorigenic activity against AML-M2 with t (8;21). FTY720 mediates apoptosis of Kasumi-1 cells in a caspase-dependent manner. In accordance with previous studies [5], we found that FTY720 treatment activated PP2A, butisonly partially responsible for the apoptosis. In order to explore the mechanism of FTY720's antitumorigenic activity in AML-M2 leukemia, a combination of microarray based bioinformatic and high-performance liquid chromatography–electrospray ionization tandem mass spectrometry (HPLC-ESI-MS/MS) lipidomic studies were carried out. It was revealed that FTY720 treatment could upregulateAML1 targeted genes, interfere with genes involved in ceramide synthesis and rapidly increase the intracellular levels of ceramide. Using small molecule inhibitors to block ceramide generation effectively reduced the pro-apoptic effect of FTY720, indicating that ceramide accumulation is a crucial event in FTY720 induced apoptosis. Furthermore, mass spectrum measurements of mitochondrial sphingolipid metabolites and simulated structural model of the ceramide and inhibitor 2 of protein phosphatase 2A (I2PP2A) complex imply that ceramide initiates caspase-dependent apoptosis machinery through directly activating mitochondria, and binding to and inhibiting I2PP2A, which results in PP2A activation and cell death. Since novel agents with new anti-tumor mechanisms for AML-M2 are in urgent demand, targeting sphingolipid metabolism by using FTY720 or FTY720 mimics may provide newinsights into the development of anti-AML drugs.

## Materials and Methods

### Cells and Chemical reagents

AML cell lines Kasumi-1 and SKNO-1 were obtained from American Type Culture Collection (ATCC, Manassas, VA). Peripheral blood mononuclear cells (PBMCs) enriched by Ficoll separation were obtained from leukemia patients and healthy donor. The protocols used in this study were approved by Rui Jin Hospital Ethics Review Boards. Written informed consents were obtained from all the patients and healthy donors in accordance with the Declaration of Helsinki. Animals were used according to the protocols approved by Rui Jin Hospital Animal Care and Use Committee. Kasumi-1 cells and PBMC were incubated in RPMI 1640 media (Gibco/Life Technologies, Carlsbad, CA) supplemented with 10% heat-inactivated fetal bovine serum and 1% antibiotic/antimycotic (FBS; Hyclone Laboratories, Logan, UT) at 37°C in an atmosphere of 5% CO_2_. FTY720 was purchased from Selleck. Caspase-3 specific inhibitor N-acetyl-Asp-Glu-Val-Asp-aldehyde (Ac-DEVD-cho), neutral sphingomyelinase (nSMase) inhibitor GW4869, and protein phosphatase 2A (PP2A) inhibitor okadaic acid (OA) were purchased from Sigma-Aldrich. N-benzyloxycabonyl-Val-Ala-Asp-fluoromethylketone (z-VAD-fmk) was purchase from R&D System. Sphingolipid standards such as C14, C16, C18, C20, and C22 ceramides were available from Toronto Research Chemicals Inc and sphingosine, S1P were purchased from Sigma.

### Cell viability analysis

Cell growth was assessed using the cell counting kit-8 (CCK8; Dojindo) assay. The cells (2×10^4^) were plated in 100 µL of media with FTY720 (ranging from 0 to 10 µM) in each well of a 96-well flat-bottomed microtiter plates in triplicate cultures and incubated at 37°C in an incubators at 5% CO_2_ atmosphere. 10 µL CCK8 solution was added to each well after46 hours treatment. The cell was culturedfor another 2 hours at 37°C. The absorbance was measured at a 450 nm wavelength with 600 nm wavelength as reference using a micro plate reader (Nanoquant; Tecan). The cell viability was expressed as a percentage of absorbance in cells with indicated treatments to that in cells of control.

### Colony formation assay

Kasumi-1 cells were plated in 24-well plates at the concentration of 4000 cells/ml with 0.5 ml IMDM medium containing 0.56% methylcellulose, 30% fetal calf serum, 1% penicillin-streptomycin, and DMSO or FTY720. Fourteen days later, colonies were counted.

### Flow cytometry assays

The cell apoptosis was determined by dual staining with annexin V conjugated to phycoerythrin (PE) and 7-amino-actinomycin (7AAD). Kasumi-1 cells or PBMCs were treated with 0, 2.5, 5, 7.5, and 10 µM FTY720 for 24 hours.Then according to the protocol, 1×10^6^ cells were stained with annexin V-PE and 7AAD (BD Pharmingen) for 15 minutes in the dark and analyzed by flow cytometry using a LSR II cytometer (BD Pharmingen). Apoptotic cells were identified as annexin V^+^ and/or 7AAD^+^ cells. Cells excluding both annexin V-PE and 7AAD were considered viable. The annexin V^−^/PI^−^ cells normalized to untreated controls are represented as a percentage oflive cells.

To evaluate effects of indicated inhibitor in FTY720-induced apoptosis, Kasumi-1 cells were incubated with indicated inhibitor for 3 h prior to treat with 7.5 µM FTY720 for 24 h. After treatment, Kasumi-1 cells were collected, and cell viability analysis was performed as described above.

### *Invivo* therapeutic efficacy evaluation in Xenograft model

The *in vivo* therapeutic efficacy evaluation of FTY720 was performed in nude mice (*nu/nu*) Xenograft model. This model was generated by subcutaneous inoculation of 3×10^7^ Kasumi-1 cells with more than 95% viability into the right flank of female nude mice 5- to 6-weeks-old. The nude mice were divided into four treatment groups when tumorsreached 100 mm^3^ approximately 10 days after inoculation. The four groups (7 or 8 mice per group) were treated with control (received placebo: 5% glucose), 5 mg/kg Ara-C (positive control), 1 mg/kg FTY720 or 5 mg/kg FTY720 per day for 3 weeks intraperitoneally. Body weight was measured every three days. The longest perpendicular tumor diameters were measured every three days, and the tumor volume was calculated by using the following formula: 0.5×(width)^2^×(length). Animals were euthanized at day 21.

Tumors excised from animals were formalin-fixed, paraffin-embedded, then the paraffin-embedded tumor sections (control, Ara-C (5 mg/kg), and FTY720 (5 mg/kg)) were used in TUNEL assay. TUNEL assay was performed to detect in situ apoptosis on tissue section using an *in situ* cell death detection kit (Roche) according to the manufacturer's protocol.

### Western blot analysis

Kasumi-1 cells were treated with FTY720, then cells were lysed with RIPA buffer (50 mM TrisHCl PH 8.0; 150 mM NaCl; 1% NP-40; 0.5% sodium deoxycholate; 0.1% SDS). Lysate with 50 µg of total protein were separated with SDS-PAGE, and transferred to PVDF membrane (GE healthcare). The blots were probed with indicated primary antibodies followed by horseradish peroxidase (HRP)-conjugated goat anti-rabbit or goat anti-mouse IgG. Detection was performed with chemi-luminescent HRP substrate (Millipore). The caspase-3, caspase-8, and caspase-9 antibodies were purchased from Cell Signaling Technology (CST). ETO antibody was purchased from Santa Cruz Biotechnology (Santa Cruz).

### PP2A activity assay

The protein phosphatase 2A activity in Kasumi-1 cells treated with or without FTY720 was determined according to PP2A immuno-precipitation phosphatase assay kit (Millipore). Kasumi-1 cells were treated with indicated concentrations of FTY720 for 24 h and thencells were pelleted and washed with ice-cold PBS. Cell pellets were incubated with phosphatase extraction buffer (20 mM imidazole-HCl, 2 mM EDTA, 2 mM EGTA, PH 7.0 with protease inhibitor cocktail and 1 mM PMSF), and then cells were subjected to sonication for 10 seconds and centrifuged at 13000 rpm for 5 min at 4°C. The protein concentration in supernatantwas quantified by BCA method. Supernatants with 100 µg protein were used to check the PP2A activity.

### Molecular Docking

The PP2A/SET protein structure was derived from the SET/TAF-1beta/INHAT complex structure (PDB: 2E50) with waters and small molecules deleted. C22-ceramide was docked to the PP2A/SET protein using Autodock Vina [18].The final docking poses were chosen based on binding free energy calculated by docking program and referring to reported studies [19], [20].

### Microarray and bioinformatics analysis

The quality of total RNA was tested by denaturing agarose gel electrophoresis. The total RNA from each sample was quantified by Nanodrop ND-1000 (GE)and RNA integrity was assessed by standard denaturing agarose gel electrophoresis. Amplification of RNA was performed with the Invitrogen Superscript ds-cDNA synthesis kit. The ds-cDNA was labeled by using NimbleGen one-color DNA labeling kit. After array hybridization using the NimbleGen Hybridization System followed by washing with the NimbleGen wash buffer kit, the array scanning was finally performed using the Axon GenePix 4000B microarray scanner (Molecular Devices Corporation). Scanned images (TIFF format) were then imported into NimbleScan software (version 2.5) for grid alignment and expression data analysis. Expression data were normalized through quantile normalization and the Robust Multichip Average (RMA) algorithm included in the NimbleScan software. All gene expression level files were imported into Agilent GeneSpring GX software (version 11.5.1) for further analysis. Genes that have values greater than or equal to lower cut-off: 100.0 in 4 out of 6 samples (“All Targets Value”) were chosen for data analysis. The microarray data was submitted to Gene Expression Omnibus (accession number: GSE55772).

Differentially expressed genes with statistical significance were identified through Volcano Plot filtering. Functional enrichment analysis of the differentially expressed genes was performed using Database for Annotation, Visualization and Integrated Discovery (DAVID) [21]. Gene set enrichment analysis (GSEA) was performed as described previously on gene lists ranked by expression level after treatment of FTY720 [22].

### Quantitative real-time PCR

For microarray analysis, total cellular RNA of Kasumi-1 cells treated or untreated with 5 µM FTY720 for 6 hours was extracted. For quantitative real-time PCR (qRT -PCR), total RNA was extracted and reverse transcribed into cDNA. qRT-PCR was performed on ABI PRISM 7900HT using SYBR Green PCR Master mix (Takara). The fold change was calculated and normalized to the transcript level of GAPDH, and the results were presented as fold change of copy number of mRNA of indicated genes. The primers used were shown in Table S1.

### Preparation of cytosol and mitochondria

Kasumi-1 cells (1×10^7^) treated or untreated with FTY720 were collected and washed with ice-cold PBS. The cell pelletswerere-suspended in 100 µl buffer (20 mM HEPES (PH 7.4), 250 mM sucrose, 5 mM MgCl_2_, 10 mMKCl, 1 mM EDTA, 1 mM EGTA, and 0.025% digitonin) and incubated on ice for 20 min. After incubation, cytosol and mitochondria fractions were prepared according to previous report [23].

### Quantization of sphingolipids by HPLC-ESI-MS/MS

Calibration curves for ceramide (C14-, C16-, C18-, C20-, and C22-ceramide), sphingosine, and sphingosine-1-phosphated were prepared by using indicated standards with concentrations ranging from 0, 10, 50, 100, 500, 1000, 5000, 10000 ng/ml. No internal standard was used in the experimental system, but all samples were prepared in the same protocol.

Kasumi-1 cell pellets (5×10^6^ cells) treated with or without FTY720 were collected and washed with ice-cold PBS. The cell pellets were sonicated 3 times periodically for 30 s after the addition of 100 µl of water. Protein content was quantified by the bicinchoninic acid (BCA) method (Beyotime Institute of Biotechnology). Lysates with 1 mg of total protein were used for sphingolipids extraction. Samples were added 1 ml of chloroform/methanol 2∶1 (v/v) and were mixed for 20 min on a rotator at room temperature, 400 µl of 0.5 M KH_2_PO_4_ was added, and samples were mixed for another 20 min on a rotator at room temperature. Samples were centrifuged at 13000 rpm for 5 min, the lower phase containing sphingolipids was collected. 1 ml chloroform was added to the remnant sample for re-extraction, the sample was mixed by vortex and centrifugated at 13000 rpm for 5 min. The lower phase was collected; the two fractions were combined and dried under a steady nitrogen stream at 40°C.

The dried residue was reconstituted with 200 µl of chloroform/methanol 1∶1 (v/v) and centrifuged for 5 min at 13000 rpm, transferred supernatant to an auto-sampler HPLC vial, injected 1 µl on Agilent HPLC system to analyze ceramide, sphingosine, and sphingosine-1-phosphate. For the ESI-MS/MS analysis, 1 mM ammonium formate in methanol containing 0.2% formic acid was served as mobile phase.

Ceramides with different carbon chain length, sphingosine, and S1P content of Kasumi-1 treated or untreated with FTY720 were calculated according to indicated calibration curves.

### Statistical analysis

All results were obtained from three separate experiments and expressed as the mean ± S.D. of data. SPSS software (version 16.0) was used for all statistical analysis. Significance was tested based on two-side P values, and all statistical analyses were performed by using paired *t*-test. *P*<0.05 level was considered significant.

## Results

### FTY720 induces apoptosisof leukemic cells from cultured Kasumi-1 cell line, Xenograft nude mouse model and AML patients

**Figure 1:**
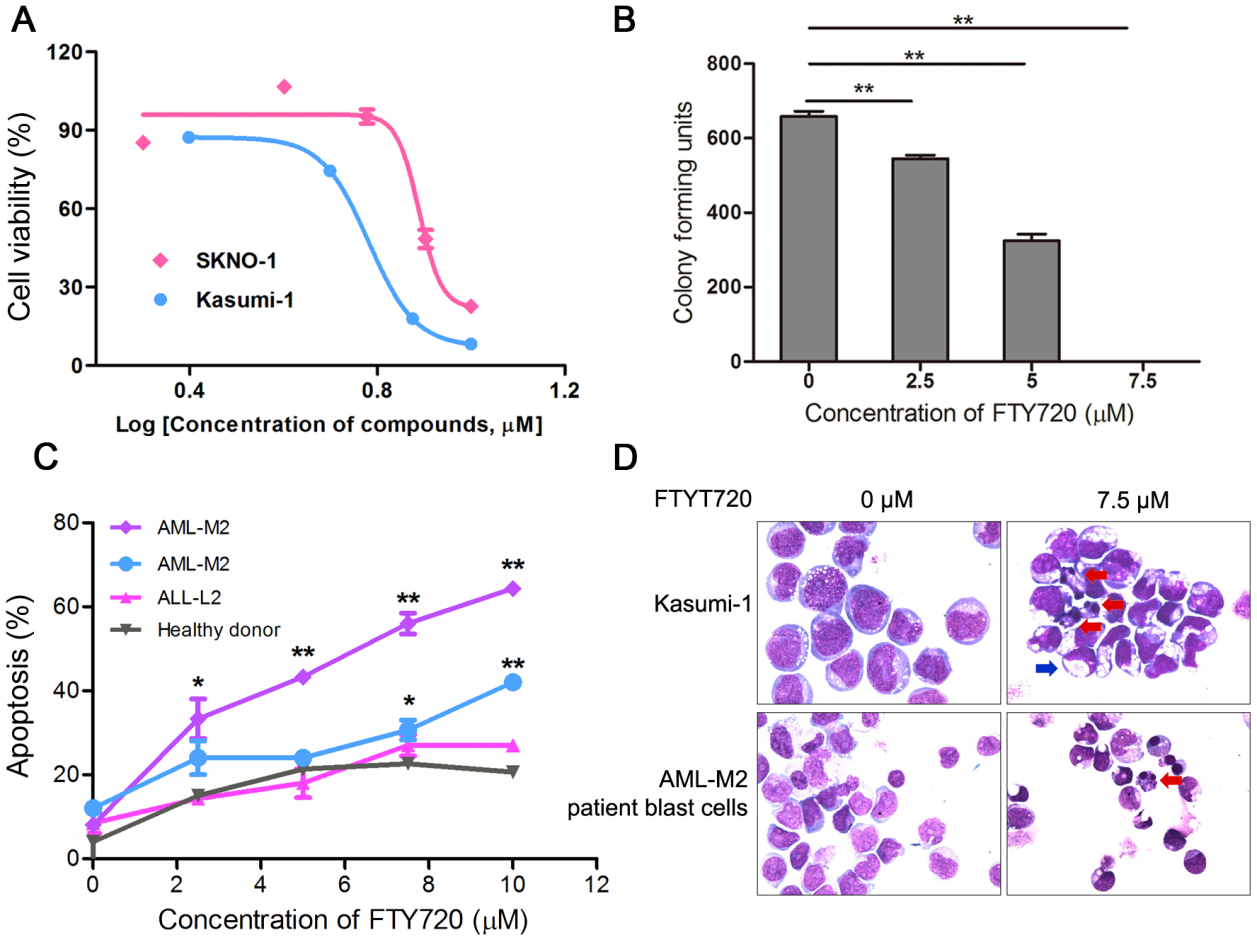
*In vitro* efficacy of FTY720 on Kasumi-1 cells and fresh leukemic cells. (A) Kasumi-1 and SKNO-1 cells were treated with indicated concentration of FTY720 for 48 h, and the cell viability was determined with a CCK-8 kit. (B) Kasumi-1 cells were grown in methylcellulose for 14 d in presence of FTY720. **P<0.01, student's *t* test compared with untreated. (C) PBMCs from leukemia patients or healthy donor were treated with or without FTY720 for 24 h, and then subjected to apoptosis analysis by flow cytometry. *P<0.05, **P<0.01. (D) Kasumi-1 (top panel) and fresh leukemic cells (bottom panel) from AML-M2 patient were treated or untreated with FTY720 (7.5 µM), and analyzed by wright staining. Partial apoptotic cells were indicated by red arrows, and blue arrow presented cells might undergo differentiation.

**Figure 2:**
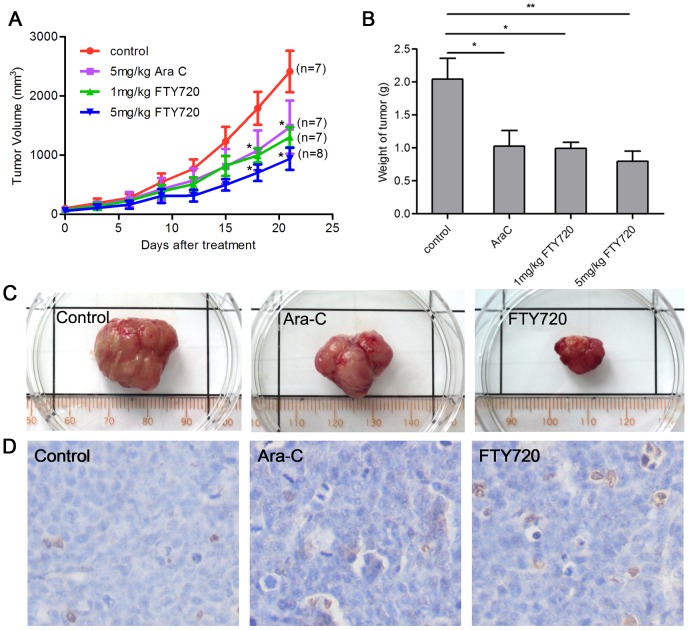
FTY720 suppresses Kasumi-1 xenograft tumor growth. Nude mice (7 or 8 mice per group) with Kasumi-1 tumor were injected intraperitoneally with vehicle (5% glucose), Ara C (5 mg/kg, positive control) or FTY720 (1 mg/kg, 5 mg/kg) for 21days. (A) Tumor volumes were measured every three days. Data are expressed as mean volumes. *P<0.05. (B, C) Animals were sacrificed at day 21, and tumors were excised and weighed. Average (B) and representative (C) results are shown. *P<0.05. **P<0.01 (D) TUNEL assay was used to detect apoptotic cells in tumors of Kasumi-1 Xenograft models treated with vehicle, Ara-C (5 mg/kg), or FTY720 (5 mg/kg).

Various studies showed that FTY720 had significant antitumorigenic activities against hematologic malignancies including acute lymphoblastic leukemia (ALL), chronic lymphocytic leukemia (CLL) [5], NK-cell leukemia [6], T-cell large granular lymphocyte (LGL) leukemia(T-LGL) [7], Blast crisis of chronic myelogenous leukemia (CML-BC) [8] and AML with c-kit mutation [9]. However, fewstudies reported the activity and mechanisms of FTY720 in M2 subtype acute myeloid leukemia (AML-M2), which is associated with t(8;21) chromosomal translocation and the resultant AML1-ETO fusion gene. Kasumi-1 cell line,which wasestablished from an AML-M2 patient harbors AML1-ETO fusion protein and Asn822Lys mutation of C-KIT protein [24]. Our initial experiments demonstrated that FTY720 inhibited proliferation and mediated apoptosis of Kasumi-1 cell line in a dose-dependent manner(IC_50_ = 5.9 µM) (Fig. 1A), and FTY720 significantly inhibited colony formation of Kasumi-1 cells *in vitro* (Fig. 1B).Kasumi-1 cells subjected to FTY720 treatment largelypresented apoptotic morphological changes with condensation and fragmentation of nuclei and cells, but only a few showed morphologic characteristics of differentiation (Fig. 1D). FTY720 also significantly inhibited proliferation of another AML1-ETO containing cell line SKNO-1 with IC_50_ of 6.2 µM (Fig. 1A). In order to evaluate the*in vivo* effect of FTY720 on AML1-ETO harbored cells, we used Kasumi-1 cell-inoculated Xenograft mouse model by subcutaneous injection of Kasumi-1 cells in nude mice. Compared to the control mice of Ara-C treatment, treatment with FTY720 at 1 mg/kg and 5 mg/kg significantly inhibited tumor growth, exerted more antitumorigenic activity than Ara-C and induced in situ apoptosis (Fig. 2A–D). In addition, we incubated blast cells from AML-M2, ALL-L2 (acute lymphoblastic leukemia) patients and healthy donors with increasing concentration of FTY720, and observed a dose-dependent increase in the percentage of the cells that underwent apoptosis identified as annexin V^+^ and/or 7AAD^+^ cells by flow cytometry (Fig. 1C). Blast cells from AML-M2 with t(8;21) patients were more sensitive to FTY720 induced apoptosis, while mononuclear cells from healthy donors were resistant to FTY720 induced apoptosis (Fig.1C–D). Taken together, FTY720 has significant anti-leukemia efficacy for AML-M2 with t(8;21).Due to the limited material available from patients, and FTY720 induced similar dose-dependent apoptotic cell death in SKNO-1 and Kasumi-1 cell lines, we used Kasumi-1 cell line throughout the remainder experiments for further mechanistic studies.

### FTY720-mediated apoptosis in Kasumi-1 cells is dependent on activation of caspases

**Figure 3:**
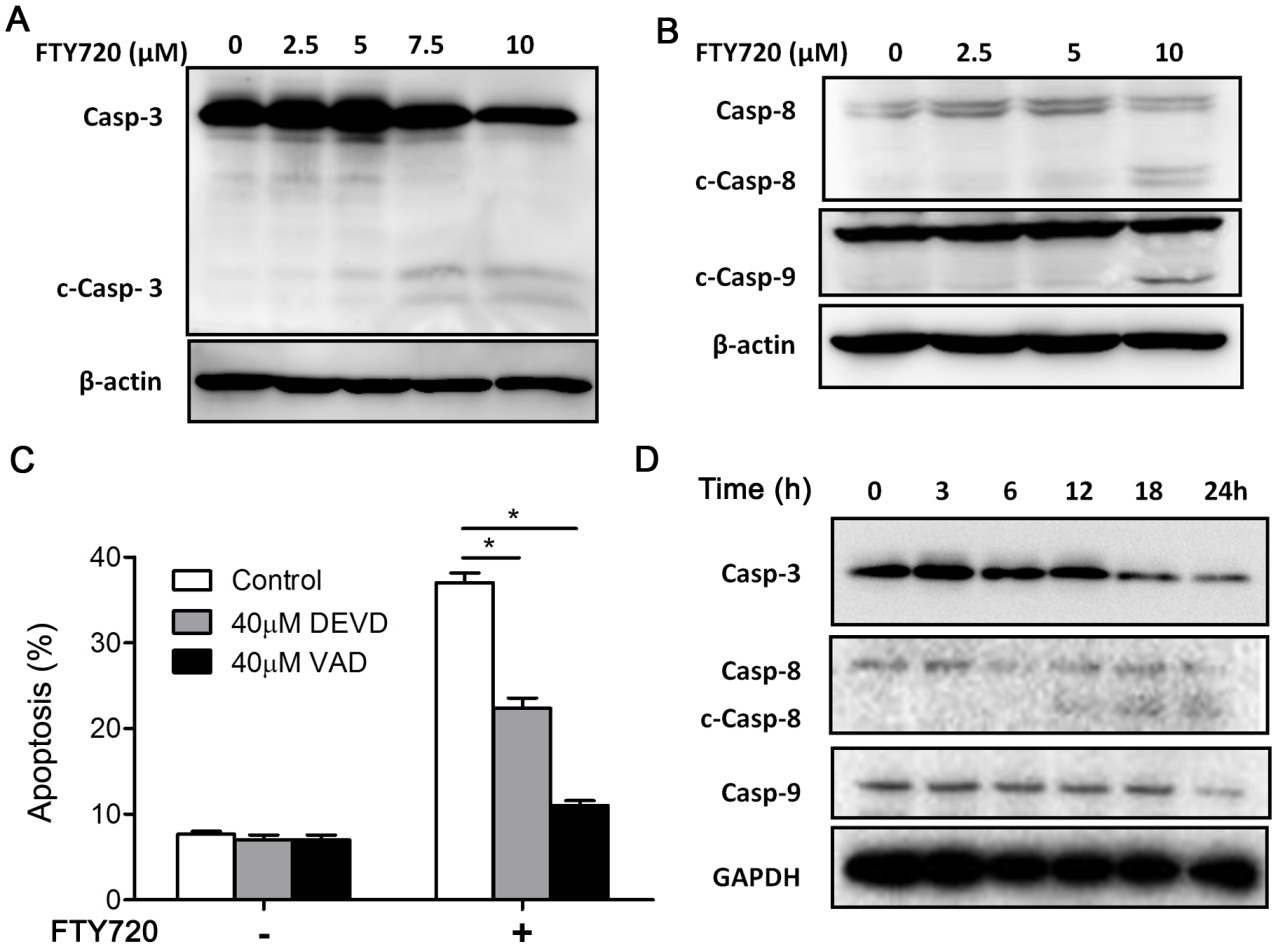
FTY720 results in caspase-dependent cell apoptosis. Western blotting assay was used to analyze the activation of caspases in Kasumi-1 cells treated with FTY720. Kasumi-1 cells were treated with indicated concentrations of FTY720 for 24 h. Caspase-3 (casp-3) and activated caspase-3 (c-casp-3) were shown by the same antibody blotting (A), caspase-8 (casp-8), active caspase-8 (c-casp-8), caspase-9 (casp-9) and active caspase-9 (c-casp-9) were analyzed by indicated antibodies blotting (B). (C) Kasumi-1 cells were pretreated with DEVD or VAD for 3 h, and subjected to 7.5 µM FTY720 for another 21 h. The effects of DEVD and VAD on FTY720-induced apoptosis were evaluated by flow cytometry. *P<0.05. (D) Kasumi-1 cells were treated with 7.5 µM FTY720 for indicated times, and then western blotting assay was carried out to analysis casp-3, casp-8, and casp-9 activation in Kasumi-1 cells treated with FTY720 for different time.

To study the mechanism of FTY720 induced apoptosis, we firstly sought to determine whether caspase activation was involved, since FTY720 has been reported to induce either caspase dependent or caspase independent cell death in leukemic cells [5], [6], [12]–[14]. We foundthat FTY720 treatment activated caspase-3, 8, 9simultaneously (Fig.3A–B). Kasumi-1 cells were pretreated with or without 40 µM Caspase-3 specific inhibitor N-acetyl-Asp-Glu-Val-Asp-aldehyde (Ac-DEVD-cho, DEVD) or a broad-spectrum caspases inhibitor, N-benzyloxycabonyl-Val-Ala-Asp-fluoromethylketone (z-VAD-fmk, VAD)for 3 hours, and then treated with 7.5 µM FTY720 for another 21 hours. DEVD partially rescued FTY720-induced apoptosis while VAD effectively blocked FTY720-mediated apoptosis (Fig. 3C). Then, we checked the time dependent manner of FTY720 (7.5 µM)-induced activation of various caspases. It was shown that all tested caspases (caspase-3, 8, 9) needed more than 12 hours of treatment to be activated. Caspase-8,caspase-3 and caspase-9 were activated in Kasumi-1 cells treated with FTY720 after12 hours, 18 hours and 24 hours, respectively (Fig. 3D).

### FTY720-mediated apoptosis partially depends on PP2A activation

**Figure 4:**
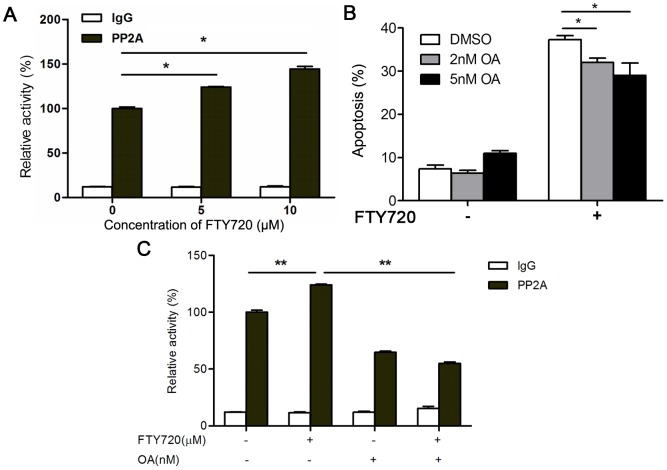
FTY720 induces Kasumi-1 apoptosis is partially dependent on PP2A. (A) Kasumi-1 cells were treated with FTY720 for 24 h, then the cell lysate was used to determine the activity of PP2A by PP2A immunoprecipitation phosphatase assay kit. *P<0.05. (B) Kasumi-1 cells were pretreated with OA for 3 h, and treated with 7.5 µM FTY720 for further 21 h. The effects of OA on FTY720-induced apoptosis were evaluated by flow cytometry. *P<0.05. (C) Kasumi-1 cells were pretreated with OA or DMSO for 3 h, and treated with 7.5 µM FTY720 for further 21 h. Then the cell lysate was used to determine the activity of PP2A according to the protocol of PP2A immunoprecipitation phosphatase assay kit. **P<0.01.

Previous studies have shown that FTY720 could induce caspase activation and apoptosis by activating protein phosphatase 2A (PP2A) [5], [8], [9], [25]. We then tested whether FTY720 induced apoptosis through activating PP2A in Kasumi-1 cells. We treated Kasumi-1 cells with different dosages of FTY720 for 24 hours, and determinedthe PP2A activity in cell lysates. The results showedFTY720 activated PP2A in a dose-dependent manner (Fig. 4A). However, 3 hours pretreatment of Kasumi-1 cells with okadaic acid (OA), a small molecule inhibitor ofPP2A,only partially inhibited FTY720-mediated apoptosis (Fig. 4B), whilethe increased activity of PP2A in Kausmi-1 cellsupon FTY720 treatment was inhibited by OA (Fig. 4C).The results suggested thatother factors and mechanisms may play more important roles in the FTY720 induced apoptosis in Kasumi-1 cells.

### Gene expression profile analysis reveals FTY720 treatment interferes with sphingolipid metabolism and upregulates AML1 target genes

**Figure 5:**
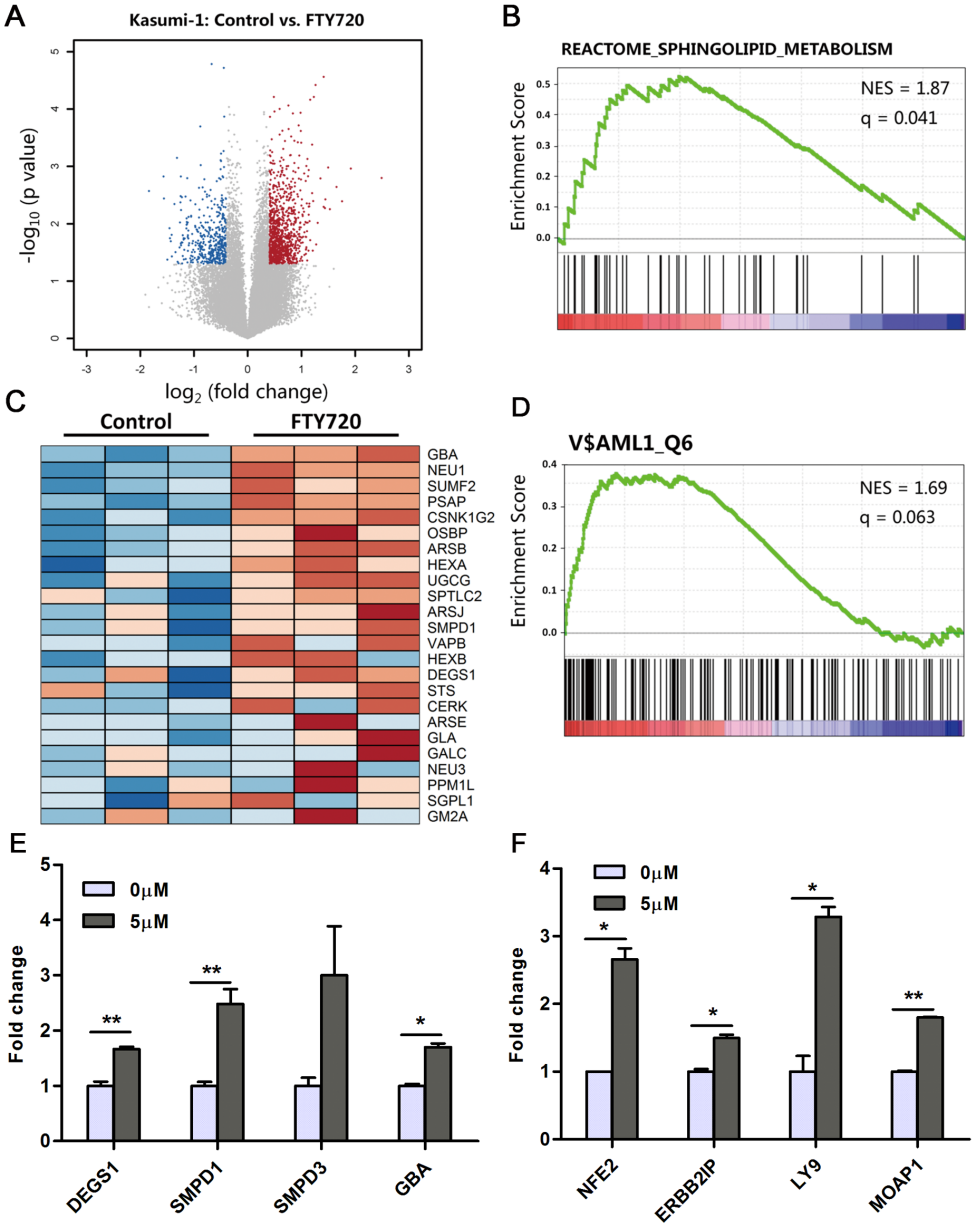
Gene expression profile analysis of FTY720 treated Kasumi-1 cells. (A) Volcano plots of gene expression differences for Kasumi-1 cells treated with FTY720 for 6 hours. Blue dots represent gene probes with *P* value <0.05 by t test and down-regulated fold change >1.5 (log_2_FC<−0.58). Red dots represent gene probes with *P*<0.05 and fold change >1.5 (log_2_FC>0.58). Gray dots represent gene probes with *P*>0.05 or fold change <1.5 (B) Gene Set Enrichment Analysis of sphingolipid metabolism signature. The enrichment is depicted by FDR q value and NES. (C) Heat map of the core enrichment genes (subset of genes that contributes the most to the enrichment result) in sphingolipid metabolism. (D) Gene set enrichment analysis of AML1 transcription factor signature. The enrichment is depicted by FDR q value and NES. (E, F) Kasumi-1 cells were treated with 5 µM FTY720 for 6 h, and RNA was extracted from cells. qRT-PCR was used to analyzetranscription of genes of ceramide synthesis pathway (E) and AML1 targets (F). *P<0.05, **P<0.01.

The molecular basis of FTY720 induced apoptosis in different type of cells was not clear and even controversial [26]. Therefore,we performed gene expression microarray profiling analysis of Kasumi-1 cells treated with 5 µM FTY720 for 6 hours, when the cells were on the early stage of reacting to the drug, to find out thedirect factors initiating FTY720 induced apoptosis. Volcano plot filtering was used to identify differentially expressed genes between control group and FTY720 treated group (Fig. 5A). According to the results, 687 genes were significantly upregulated and 277 genes were significantly downregulated after treatment of FTY720 (fold change≥1.5, *P*value≤0.05). Functional annotation clustering of these differentially expressed genes using the DAVID annotation database [21] indicatedthat the set of up-regulated genes were highly enriched in functional groups that related to cellular metabolism such as sterol biosynthetic process (*P* value  = 3.2E-7) and lipid biosynthesis (*P* value = 5.7E-5). This effect may be a protective reaction of AML cells when treated with apoptosis-inducing agents [27]. Further, results from Gene Set Enrichment Analysis (GSEA) suggested thatsome genes involved in sphingolipid metabolism were upregulated upon treatment of FTY720 (Fig. 5B). Among them, the genes involved in *de novo* salvage pathways of ceramide synthesis, such as acid sphingomyelinase (aSmase, SMPD1) [28], acid beta-glucosidase (GBA) [29]and delta (4)-desaturase sphingolipid 1 (DEGS1) [30], were shown to be upregulated by treatment of FTY720 (Fig. 5C). Ceramide is a sphingosine-based lipid signaling molecule and has been considered to be tightly correlated with programmed cell death [31].Thus, the upregulation of genes encoding enzymes involved in ceramide synthesis may be a new mechanism of FTY720 induced Kasumi-1 apoptosis.

In addition, the upregulated genesofFTY720 treatment were shown to be highly enriched in the set of downstream targets of AML1 transcription factor (*P* value = 3.68E-10) (Table S2). In accordance, GSEA using the transcription factor target sets(c3.tft.v3.1.symbols.gmt) also indicated the upregulated genes were enriched in the set of target genes of AML1 (Fig. 5D).

The qRT-PCR experiments proved that treatment with FTY720 upregulated multiple genes belonging to ceramide biosynthesis pathway such as DEGS1, SMPD1 and GBA (Figure 5E). qRT-PCR assays also revealed that neutral sphingomyelinase2 (nSmase2, SMPD3), another key enzyme in the salvage pathway of ceramide synthesis [32], was up-regulated following FTY720 treatment (Fig. 5E). The qRT-PCR assay also validated that a well-defined target gene of AML1, NFE2 [33], was upregulated after treatment of FTY720. Other genespreviouslysuggested as AML1 target genes by Chip-Seq experiment [34], [35], such as Erbb2 Interacting Protein (ERBB2IP), Lymphocyte Antigen 9 (LY9) and Modulator of Apoptosis 1 (MOAP1), were also shown to be upregulated upon treatment of FTY720 (Fig. 5F). The upregulation of AML1 target genes indicated that the function of AML1, which was blocked by the AML1-ETO fusion protein [1], [36], was partially rescued upon treatment of FTY720. Restoration of AML1 function may further reduce tumor growth and contribute to enhancing potency of FTY720 in Kasumi-1 cells.

### FTY720 causes AML1-ETO oncoprotein elimination and G_0_/G_1_ cell cycle arrest

**Figure 6:**
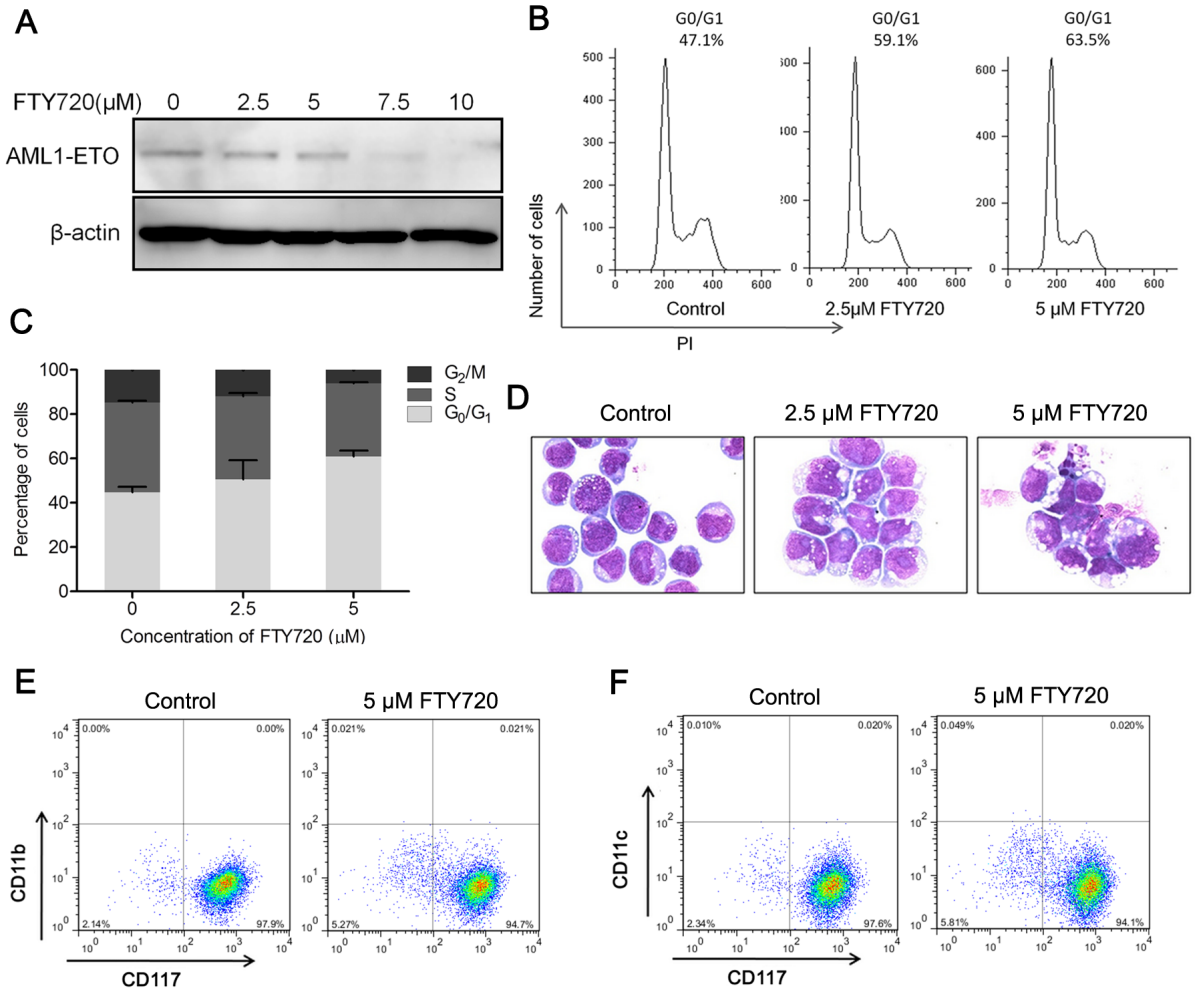
FTY720 induces elimination of AML1-ETO oncoprotein and causes cell cycle arrest. (A) Kasumi-1 cells were treated with indicated concentrations of FTY720 for 24 h, and cell lysate was subjected to western blotting analysis. Anti-ETO antibody was used to detect AML1-ETO fusion protein. (B) Kasumi-1 cells were treated with FTY720 for 72 hours, and cell cycle of Kasumi-1 cells treated FTY720 was analyzed by flow cytometry after propidium iodide (PI) staining, representative histograms were shown. (C) Cell cycle progression of Kasumi-1 treated with FTY720 was evaluated, nuclei stained with PI was used to DNA content analysis by flow cytometry. (D) Morphologic changes in Kasumi-1 treated for 3 days with indicated concentrations of FTY720. (E, F) Quantification of CD11b (E), CD11c (F), and CD117 expression in Kaumi-1 cells treated for 3 days with FTY720 was detected with flow cytometry.

As gene expression profiling results indicated FTY720 could reverse AML1-ETO mediated transcriptional repression, we performed western blot assays to investigate whether the expression of AML1-ETO oncoprotein was affected by FTY720. We saw a dramatic elimination of AML1-ETO fusion protein in Kasumi-1 treated with 7.5 µM or higher dosage of FTY720 for 24 hours (Fig. 6A). AML1-ETO could repress AML1 target genes and block the differentiation of hematopoietic stem cells [34], [36]. Studies have shown that siRNA-mediated depletion of AML1-ETO in Kasumi-1 cells led to a sensitization towards myeloid differentiation and increase of the G_1_/G_0_ fraction [1]. As mentioned above, a few of FTY720 treated Kasumi-1 cells presented morphological characteristics of differentiation. We then further evaluated the differentiation of Kasumi-1 cells treated with FTY720 by following criteria: cell cycle arrest in G_1_ phase, and expression of differentiation markers. DNA content showed an increment of the percentage of cells in G_0_/G_1_ phase after incubation with FTY720 for 48 hours (Fig. 6B–C). However, we failed to detect any expression changes of CD117, CD11b and CD11c, three differentiation markers for hematopoietic stem cells, after FTY720 treatment for 72 hours (Fig. 6E–F), while signs of apoptosis were significant (Fig. 6D). The cell cycle results were similar to previous report that FTY720 could induce G_0_/G_1_ cell cycle arrest of human lymphoma cell lines [37].Taken together, these results suggested that FTY720 could eliminate AML1-ETO fusion protein and induce cell cycle arrest. However, the cells underwent apoptosis before showing detectable sign of differentiation. Therefore, another dominant mechanism forFTY720's effect on Kasumi-1 cells may exist.

### FTY720 induces the accumulation of ceramide in Kasumi-1 cells

**Figure 7:**
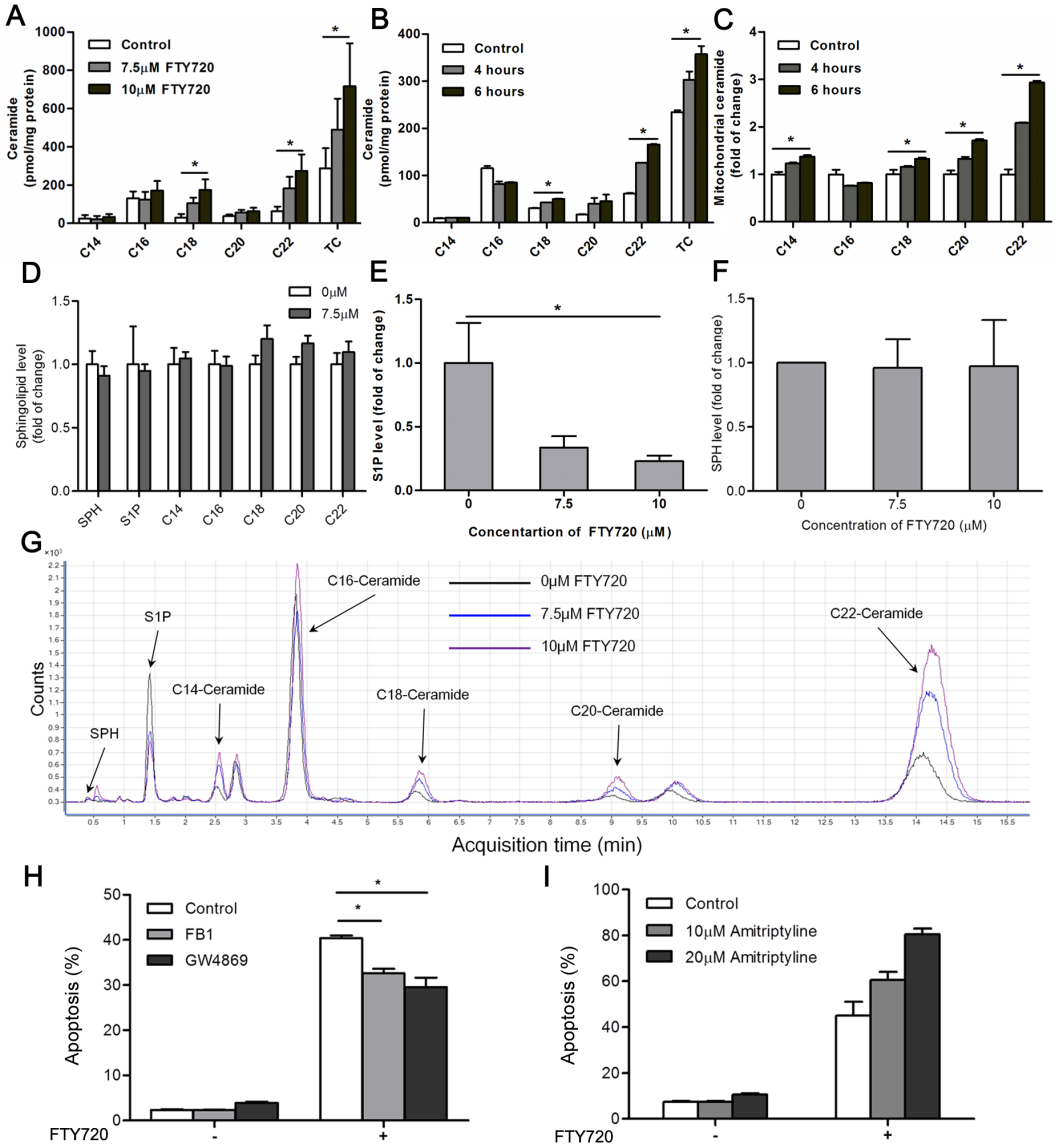
FTY720 induces Kasumi-1 cell apoptosis primarily through elevating ceramide levels. (A, B) Ceramide levels in Kasumi-1 cells treated FTY720 with indicated concentrations (A) and different times (B) were determined by HPLC-ESI-MS/MS assay, TC means total ceramide (C14-, C16-, C18-, C20-, and C22-Ceramide). *P<0.05. (C) Kasumi-1 cells were treated with FTY720 for indicated times, and mitochondria was separated. The contents of ceramide in mitochondria of FTY720 treated Kasumi-1 cells were determined by HPLC-ESI-MS/MS assay. * P<0.05. (D) Kasumi-1 cells treated with or without Ara-C (7.5 µM) for 24 h, the contents of sphingolipid were determined by HPLC-ESI-MS/MS assay. (E, F) Kasumi-1 cells treated with indicated concentrations of FTY720 for 24 h, the contents of S1P (E) or SPH (F) were determined by HPLC-ESI-MS/MS assay. *P<0.05. (G) Kasumi-1 cells treated with indicated concentrations of FTY720 for 24 h, the signals of sphingolipids detected by HPLC-ESI-MS/MS assay were shown. (H) Kasumi-1 cells were pretreated with 20 µM FB1 or GW4869 for 3 h, and treated with 7.5 µM FTY720 for further 21 h. The effects of FB1 or GW4869 on FTY720-induced apoptosis were evaluated by flow cytometry. *P<0.05. (I) Kasumi-1 cells were pretreated with amitriptyline for 3 h, and treated with 7.5 µM FTY720 for further 21 h. The effects of amitriptyline on FTY720-mediated apoptosis were determined by flow cytometry.

Since the upregulation of AML1 target genes may not be the major effects of FTY720 induced apoptosis and our gene expression profiling demonstrated that FTY720 disturbed the sphingolipid metabolism and could activate ceramide synthesis pathway, we used HPLC-ESI-MS/MS analysis to monitor the changes of three functional sphingolipid metabolites - ceramide, sphingosine, and sphingosine-1-phosphate (S1P) in Kasumi-1 cells treated with or without FTY720 for 24 hours. The contents of ceramide C18, C20 and C22 were increased in Kasumi-1 cells treated with FTY720 in a concentration-dependent manner (Fig. 7A, G). Significant accumulation of ceramide was detected as early as 6 hours after treatment, when cells showed normal morphology and were negative for 7AAD and Annexin V staining (Fig. 7B). However, 7.5 µM of Ara-C treatment did not alter the cellular level of sphingolipids in Kasumi-1 cells (Fig. 7D). On the other hand, FTY720 treatment decreased cellular S1P in a dose dependent manner but had no significant effects on cellular level of sphingosine (SPH) (Fig. 7E–F).

### Accumulation of ceramide plays a central role in FTY720 induced apoptosis

Since alterations of ceramide metabolism after FTY720 treatment were revealed by microarray and HPLC-ESI-MS/MS assay, it was necessary to investigate the role of ceramide in FTY720 induced apoptosis signaling pathway. We pretreated Kasumi-1 cells with 20 µM GW4869, a neutral sphingomyelinase (nSMase) inhibitor [38] for blocking the salvage pathway of ceramide generation, prior to FTY720 treatment. As expected, GW4869 significantly reduced FTY720-mediated apoptosis (Fig. 7H). In addition, using a ceramide synthases inhibitor Fumonisin B1 (FB1, 20 µM), which could block the *de novo* pathway of ceramide synthesis [39], also partly rescued Kasumi-1 cells from FTY720 induced apoptosis. However, pretreatment with amitriptyline, an acid sphingomyelinase (aSmase) inhibitor, failed to protect Kasumi-1 cells from FTY720-mediated apoptosis (Fig. 7I). Previous studies indicated that the contribution of lysosomal aSmase was limited in stress-induced cell death [40]. Therefore, we suggest that, although we have seen an upregulation of aSmase gene *SMPD1* in microarray and qRT-PCR assays, aSmase may not take part in FTY720 induced apoptosis in Kasumi-1 cells, while neutral sphingomyelinases, such as nSmase2, and enzymes for *de novo* ceramide synthesis may play adominant role in FTY720-mediated ceramide generation and apoptosis.

Studies suggested that ceramide could target mitochondria directly and lead to mitochondria outer membrane permeabilization (MOMP), resulting in caspase activation and apoptosis, and mitochondrial ceramide levels increasing prior to the mitochondrial stage of apoptosis [41]. Thus, we extractedthe mitochondria of Kasumi-1 cells treated or untreated with FTY720, and used HPLC-ESI-MS/MS to detect ceramide levels on mitochondria. Same as the total cellular ceramide accumulation, HPLC-ESI-MS/MS analysis revealed a significant increase of mitochondrial ceramide, especially C22-ceramide, as early as 4 hours after FTY720 treatment (Fig. 7C).

**Figure 8:**
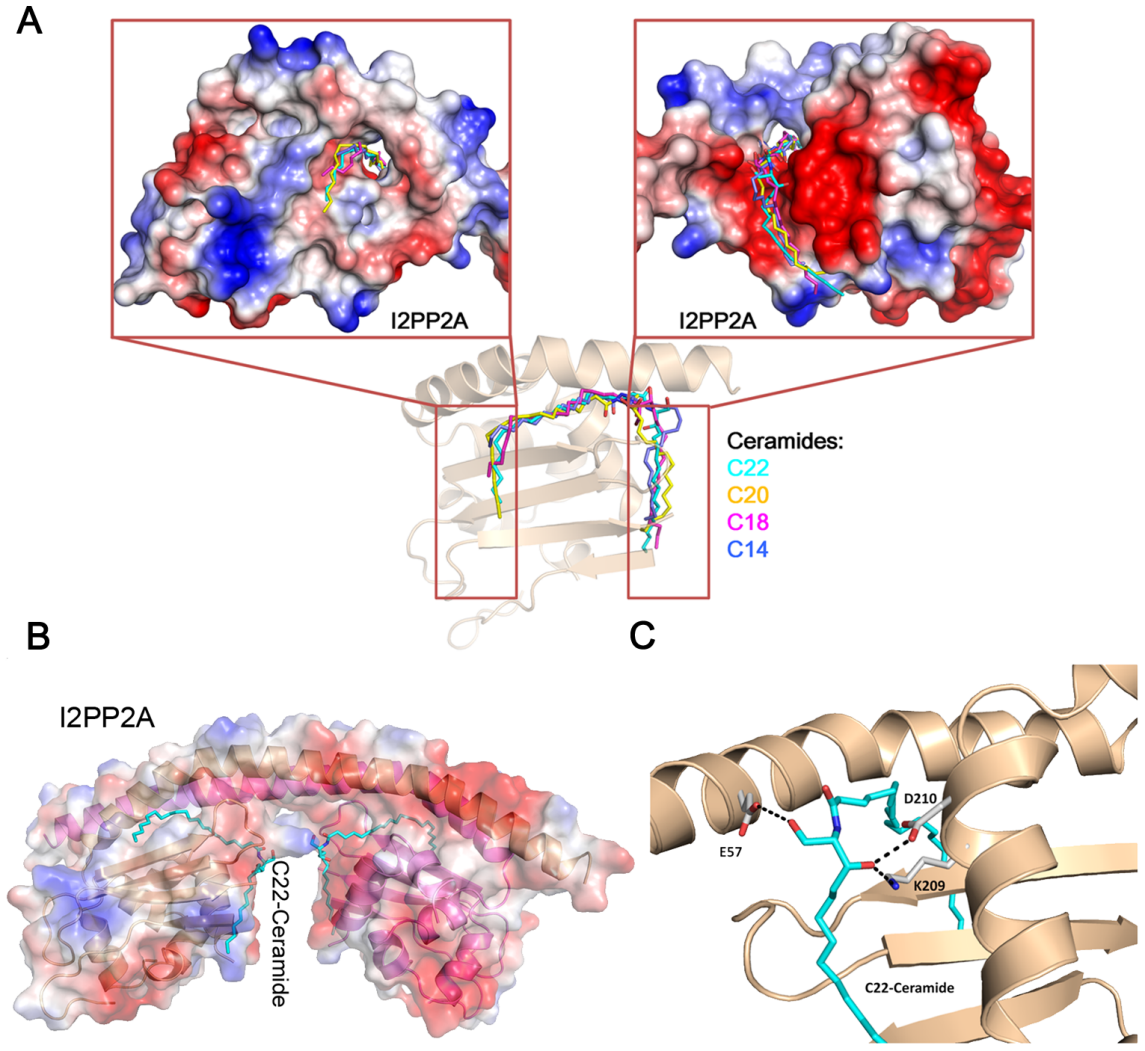
Binding of ceramides to I2PP2A/SET. (A) Docking of C22-ceramide to I2PP2A/SET. The surface contour was colored by electrostatic potential. The atoms of C22-ceramide were shown as sticks. (B) The binding mode of C22-ceramide with I2PP2A/SET. C22-ceramide and the residues involved in the interactions with C22-ceramide are shown as sticks. The hydrogen bonds formed between C22-ceramide and I2PP2A/SET were shown as black dashes. (C) Binding modes between I2PP2A/SET and ceramide with various chain lengths predicted by molecular docking. Front and rear direction views show the bond ceramide composed sphingosine and fatty acid chains.

Since our experiment indicated that FTY720 induced apoptosis only partially depended on the PP2A activation, we thought to seek the direct factors responsible in Kasumi-1 cells. We noticed a newly released study in A549 human lung cancer cells identified the inhibitor 2 of PP2A (I2PP2A/SET) as a ceramide-binding protein [25], [42]. FTY720 and ceramide both directly bind to I2PP2A/SET with a high affinity (K_d_ ∼10 nM). As I2PP2A/SET is an endogenous inhibitor of PP2A, I2PP2A/SET inhibition by ceramide binding could lead to PP2A activation and cell death [25], [42]. During our current studies in AML leukemia cells, we observed PP2A activation and a significant accumulation of cellular ceramide following FTY720 treatment. Therefore, we docked ceramide species that increased significantly following FTY720 treatment, i.e. C18-, C20- and C22-ceramide, to the dimmerized “headphone”-like structure of I2PP2A/SET (PDB: 2E50), which is the biologically functional conformation inside the cell. It is interesting that the ceramide lies on the bottom surface of the I2PP2A/SET “earmuff” domain, and thisregion is engaged in histone chaperone activity for binding to core histones and double-stranded DNA [43]. All ceramides adopted similar docking poses and bound to the hydrophobic concave, whichcould accommodate thevarious chain lengths of ceramide (Fig. 8A) [25], [42]. Figure 8B and Figure 8C showedthe detailed binding pose between C22-ceramide and I2PP2A/SET. The simulated binding mode between C22-ceramide and I2PP2A/SET suggested that E57, K209, D210 form three hydrogen bonds with two hydroxyl groups of C22-ceramide (Fig. 8C). The role of K209 in I2PP2A/SET-ceramide interaction has been validated by reported mutational studies [25], [42].

Taken together, these results indicate that FTY720 induces apoptosis of Kasumi-1 cells mainly through accumulation of ceramide, and that ceramide may initiate apoptosis machinery through directly activating mitochondria and binding to I2PP2A/SET, resulting in PP2A activation and apoptosis.

## Discussion

We showed that FTY720, an FDA approved oral drug, could induce apoptosis of Kasumi-1 cells, an AML cell line possessing both t(8;21)(q22:q22) and c-kit mutations, *in vitro* and in Xenograft model. FTY720 showed higher efficacy than currently used chemotherapy agent, Ara-C. In addition, FTY720 induced apoptosis of blast cells from AML patients in a dose dependent manner and showed selectivity for blast cells from AML-M2 patients. Given the high potency of inducing apoptosis of AML cells and low toxicity towards normal cells, FTY720 or FTY720 mimic may provide novel insight into drug development for AML-M2 treatment. Considering that our initial findings of the caspase activation is a terminal event in programmed cell death, and thatPP2A activation is only partially responsible for FTY720 induced apoptosis, we sought to discover the dominant factors controlling the mechanism of FTY720 induced apoptosis. Through a combined bioinformatic and lipidomic study, we identified a perturbation of sphingolipid metabolism pathway by FTY720 treatment, which ledto the accumulation of ceramide- a pro-apoptotic second messenger. Microarray and qRT-PCR studies indicated that enzymes encoded by some of the upregulated genes, such as DEGS1, SMPD1, SMPD3, and GBA, were involved in the ceramide synthesis pathway, which indicated that FTY720 treatment might elevate the endogenous ceramide levels. In accordance with microarray and qRT-PCR results, HPLC-ESI-MS/MS analysis revealed a significant accumulation of ceramide in Kasumi-1 cells following FTY720 treatment.On the other hand, FTY720 could upregulate AML1 target genes and result in an increase of G_1_/G_0_ fraction in Kasumi-1 cell.Thecellstreated with FTY720 might partially undergo differentiation; however, the endogenous ceramide accumulation ultimately dictates cell death by apoptosis. Our current study revealed that ceramide related molecular events might be the upstream and dominant effect behindthe mechanism of FTY720 induced apoptosis in Kasumi-1 cell.

Although FTY720, which is a sphingosine mimic, is structurally similar to ceramide, the role of ceramide in FTY720 induced apoptosis has rarely been studied. However, the role of ceramide as a second messenger in cellular apoptosis has been well characterized [31]. In the present study, we have shown that FTY720 treatment significantly and rapidly increased endogenous ceramide of various chain lengths. Ceramide levels in FTY720-treated Kasumi-1 cells were elevated as early as 4 hours, when caspases were inactive and apoptotic markers were negative.Ithas been known that ceramide is played greater potency than FTY720 in PP2A activation [44], [45], and ceramide could also increase the permeability of the mitochondrial outer membrane directly, releasing pro-apoptotic proteins such as cytochrom C and eventually led to caspase activation and apoptosis [41]. Although the mitochondrial preparation may contain endoplasmic reticulum(ER), we observed a significant accumulation of ceramide on mitochondrial membrane after FTY720 treatment. Therefore, we suggest that apoptosis induced by FTY720 largely owes to ceramide accumulation after FTY720 treatment.

The precise mechanism of how FTY720 perturbs the sphingolipid metabolism and leads to ceramide accumulation remains elusive. A study on prostate cancer cells has shown that FTY720 could moderately elevate cellular ceramide levels by directly inhibiting SPHK1 [11]. In addition, Steven et al. have shown that a SPHK1 specific inhibitor named SKI-1 could induce apoptosis of U937 cells [46]. In their study, SKI-1 inhibited SPHK1 and caused ceramide increasing and S1P decreasing. Although decrement slight decrease of cellular S1P after FTY720 treatment was observed, we would exclude the possibility that the accumulation of ceramide in Kasumi-1 cells resulted from the direct inhibition of SPHK1 by FTY720, asthe amount of increased ceramide far exceeded the amount of decreased S1P and we did not observe increase of sphingosine after FTY720 treatment, as reported in prostate cancer cells [11] and NK-cell leukemia [6]. Thus, the majority of increased ceramide must come from other sources such as salvage or *de novo* ceramide synthesis (Figure S1). Furthermore, it was reported that FTY720 could increase the generation of long chain ceramide in part through the salvage pathway and fumonisin B1 treatment also partially rescued cells from FTY720-induced death [15]. However, as a multi-target drug, it's difficultto rule out the ability of FTY720 inhibition to SPHK1 contributes to its cytotoxicity on Kasumi-1 cells, because reducing the cellular level of anti-apoptotic S1P could further sensitize tumor cells to FTY720 and ceramide induced apoptosis [47]. In addition, the role of nSmase2 in stress-induced ceramide generation has been well recognized [32] and nSmase2 gene is frequently mutated or downregulated in acute leukemia [48], [49]. Therefore, combining our qRT-PCR assays and chemical inhibitor experiment, we suggest nSmase2 might be crucial for the ceramide accumulation and cell death induced by FTY720.

In conclusion, our studies demonstrated that FTY720 exerted potent antitumorigenic effects on Kasumi-1 cells, both in animal models, as well as in blast cells derived from AML-M2 patients. Our data indicated FTY720 treatment could activate ceramide synthesis pathway, which in turn leads to the increase of ceramide in Kasumi-1 cells, particularly on mitochondria membrane. Endogenous ceramide activates PP2A or targets mitochondria to induce MOMP, which eventually activates caspase cascade and initiates apoptosis machinery. In addition, the elimination of AML1-ETO may still facilitate FTY720 induced apoptosis of Kasumi-1 cells by mediating cell cycle arrest, although it failed to induce cell differentiation. Collectively, these data demonstrate that FTY720 may provide a novel insight into drug development for AML-M2 treatment and targeting sphingolipid metabolism may also be a promising way to treat AML-M2.

## Supporting Information

Figure S1 **Metabolic pathways of sphingolipids.**
(DOCX)

Table S1 **Primers of indicated genes used in RT-PCR.**
(DOCX)

Table S2 **Top 10 enriched transcription factors identified by functional annotation clustering using DAVID database.**
(DOCX)
