# Supplementary figures and images for: FTY720 Induces Apoptosis of M2 Subtype Acute Myeloid Leukemia Cells by Targeting Sphingolipid Metabolism and Increasing Endogenous Ceramide Levels
Source: PLoS One. 2014 Jul 22;9(7):e103033. doi: 10.1371/journal.pone.0103033 (PMC4106898; doi:10.1371/journal.pone.0103033)

**Fig. S1**

**
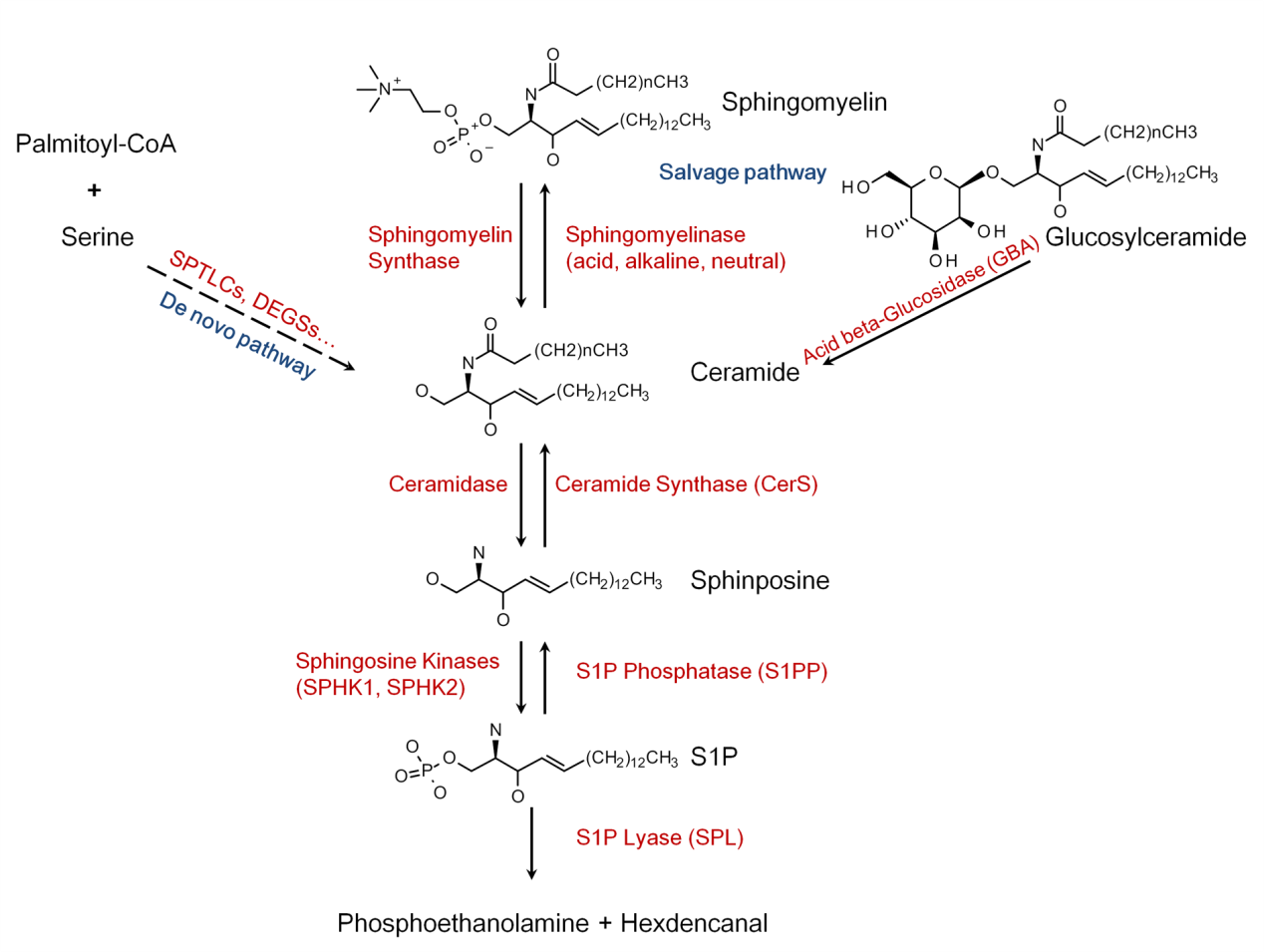
**

Supplement: Figure S1 — Metabolic pathways of sphingolipids. (DOCX) [file pone.0103033.s001.docx]
