# Supplementary material for: FTY720 Induces Apoptosis of M2 Subtype Acute Myeloid Leukemia Cells by Targeting Sphingolipid Metabolism and Increasing Endogenous Ceramide Levels
Source: PLoS One. 2014 Jul 22;9(7):e103033. doi: 10.1371/journal.pone.0103033 (PMC4106898; doi:10.1371/journal.pone.0103033)
Supplement: Table S1 — Primers of indicated genes used in RT-PCR. (DOCX) [file pone.0103033.s002.docx]

**Table S1**. Primers of indicated genes used in RT-PCR.

| Gene Symbol | Forward Primer | Reverse Primer |
| --- | --- | --- |
| CSF2RA | CCTTCTGCTCTGTGAGTTACC | GGTTGTGTTTTCTTGGCAGTC |
| NFE2 | GCAGGAACAGGGTGATACAG | TCACTTGGAGCATTCAGACC |
| ERBB2IP | GGCAAAACAAGAGATTCGAGTG | TTTGATGCTGGTCCTTCAGG |
| LY9 | CATTTGGAAGCGAAAAGGACG | CTTGGGAGAGCACAGAGTATAG |
| MOAP1 | AGGATGAGGAAAAGTTGTCGG | TGCGAATTGTTTTGTGGACTG |
| DEGS1 | CTAGTCTGCAAGCCACCG | TGGGATCAGGTTTCATCAAGG |
| SMPD1 | TCTATTCACCGCCATCAACC | TCCACCATGTCATCCTCAAAG |
| SMPD3 | AGCTCTGTTTCTCAAGGTGC | CAGCCAGTCCTGAAGCAG |
| GBA | CATACTGTGACTCCTTTGACCC | CCGTGTGATTAGCCTGGATG |
| HMGCS1 | CGGTATGCCCTGGTAGTTG | TGCATATGTGTCCCACGAAG |
| LSS | AGATAACCCTCCCGACTACC | TGCAGTCAGAAACGATCCAG |
| LDLR | TTCACTCCATCTCAAGCATCG | ACTGAAAATGGCTTCGTTGATG |
| GAPDH | AATCCCATCACCATCTTCCAG | AAATGAGCCCCAGCCTTC |
