# Supplementary material for: FTY720 Induces Apoptosis of M2 Subtype Acute Myeloid Leukemia Cells by Targeting Sphingolipid Metabolism and Increasing Endogenous Ceramide Levels
Source: PLoS One. 2014 Jul 22;9(7):e103033. doi: 10.1371/journal.pone.0103033 (PMC4106898; doi:10.1371/journal.pone.0103033)
Supplement: Table S2 — Top 10 enriched transcription factors identified by functional annotation clustering using DAVID database. (DOCX) [file pone.0103033.s003.docx]

**Table S2.** Top 10 enriched transcription factors identified by functional annotation clustering using DAVID database

| TFs | Count | P Value | FDR |
| --- | --- | --- | --- |
| AML1 | 504 | 3.68E-10 | 4.52E-07 |
| TATA | 369 | 1.45E-09 | 1.78E-06 |
| GFI1 | 313 | 2.20E-09 | 2.70E-06 |
| GATA1 | 519 | 3.10E-09 | 3.81E-06 |
| PAX2 | 393 | 5.58E-09 | 6.85E-06 |
| NKX25 | 421 | 6.92E-09 | 8.50E-06 |
| MEIS1BHOXA9 | 345 | 7.21E-09 | 8.86E-06 |
| SRY | 295 | 1.01E-08 | 1.24E-05 |
| FREAC4 | 319 | 1.05E-08 | 1.29E-05 |
| SREBP1 | 402 | 1.18E-08 | 1.46E-05 |
